# Preparation of a Work on “New Remedies”

**Published:** 1862-02

**Authors:** 


PREPARATION OF A WORK ON "NEW REMEDIES."—In connection with Prof. Percy, the undersigned is preparing a work on "New Remedies." It is based on the celebrated Essay of Dr. V. Guibert, to which the *Societe des Sciences Medicales et Naturales de Bruxelles* awarded its full prize, and the erudite elaboration thereof by Dr. Richard Hagen, now in course of publication at Leipzig, Germany. Our work will embrace all valuable medicinal agents introduced into the Treatment of Disease since the year 1830, up to the present day, detailing their history, description, action and uses, and giving the most approved Formulæ of Preparation, Preservation, and Administration. In Formulæ it will be particularly full, for the use of both physicians and pharmaceutists. Novelty notbeing deemed a sufficient passport for admission into confidence unless sustained by merit, and with the only object—*to be useful*—and the only means—*Labor to approach the Truth*—constantly before us, we are determined that no really useful remedy, introduced during the last thirty years, shall be slighted, while no undue prominence shall be given to undeserving articles Nor will uncertainty or ignorance at any point that the actual advance. of science has not reached, be sought to be concealed by illusory hypothesis or ill-founded statements. Any heretofore unpublished information calculated to add to the practical utility of the work, that may be in the possession of any of our readers, will be gratefully received, carefully considered, and, if used, appropriately acknowledged. The lamentable circumstances that at present occupy the mind of every good citizen—our beloved country's trials—may somewhat delay the realization of our enterprise; but we bespeak for it, even in these troublous times, the co-operation of the profession.

ELSBERG.

